# New-Onset Refractory Status Epilepticus (NORSE) as a Recurrence of Anti-Neuronal Nuclear Antibody 2 (ANNA-2) Encephalitis After Immune Checkpoint Inhibition Therapy

**DOI:** 10.7759/cureus.16074

**Published:** 2021-06-30

**Authors:** Danielle Pitter, Luis Mejico, Julius G Latorre, Carolina Cuello-Oderiz

**Affiliations:** 1 Neurology, State University of New York Upstate Medical University, Syracuse, USA; 2 Neuro-intensive Care/Stroke Neurology, State University of New York Upstate Medical University, Syracuse, USA

**Keywords:** status epilepticus, opsoclonus-myoclonus, encephalitis, paraneoplastic, anna-2 antibody, active immunotherapy

## Abstract

Paraneoplastic encephalitis from anti-neuronal nuclear antibody 2 (ANNA-2), usually associated with breast cancer, can cause seizures. We report a case of recurrent paraneoplastic encephalitis due to ANNA-2 presenting with new-onset refractory status epilepticus (NORSE) one month after receiving checkpoint inhibitors therapy.

A 69-year-old female was diagnosed with opsoclonus myoclonus syndrome (OMS) secondary to ANNA-2, which led to a diagnosis of breast cancer. OMS improved with surgical resection and intravenous immunoglobulin (IVIG). Three years later, she was diagnosed with metastatic cancer to the liver and spine. She underwent immune checkpoint inhibitor therapy. One month later, she was admitted with NORSE. Opsoclonus was seen at the physical exam. Brain MRI and infectious work-up were unremarkable. Cerebrospinal fluid (CSF) analysis revealed pleocytosis with lymphocytic predominance. She was treated with corticosteroids and immunoglobulins, and she had symptomatic improvement. ANNA-2 test was positive in a lower titration than three years earlier.

Opsoclonus in a patient with NORSE can be the hint of ANNA-2 positivity. Immune checkpoint inhibitor therapy should be carefully reconsidered in patients with a history of paraneoplastic encephalitis for ANNA-2 as it could precipitate NORSE.

## Introduction

New-onset refractory status epilepticus (NORSE) is a refractory status epilepticus in a patient without a history of seizures [1]. Causes are divided into infectious, toxic, autoimmune, or paraneoplastic [2]. About half of the cases remain cryptogenic even after extensive workup [2]. Anti-neuronal nuclear antibody 2 (ANNA-2) or anti-Ri, mainly linked with breast adenocarcinoma, has been associated with neurological syndromes, among which opsoclonus myoclonus syndrome (OMS) is the most frequently reported [3]. OMS can be recurrent [4]. In a case series of 34 patients who were positive for ANNA-2 and had neurological symptoms, only one patient had seizures [3]. We report the first patient with a history of OMS who presented with NORSE.

## Case presentation

**Figure 1:**
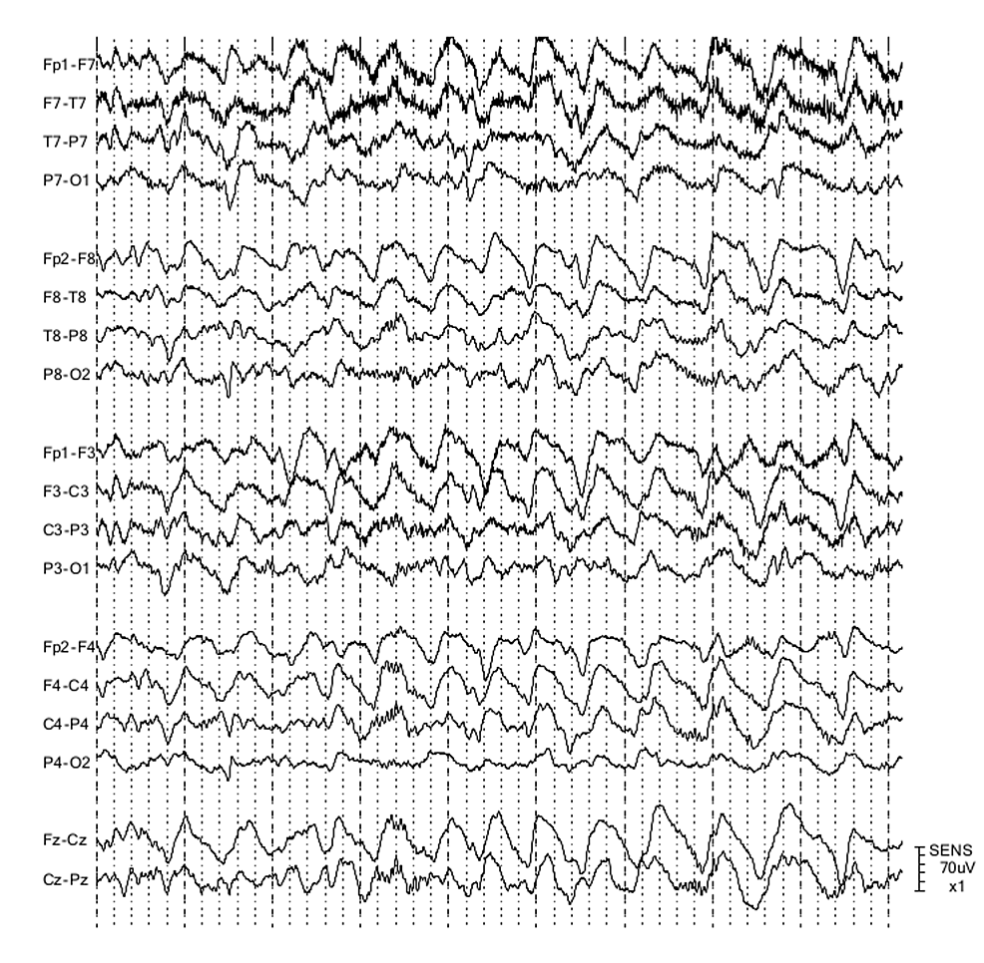
Electroencephalogram (EEG) sample during the first day of admission Double banana montage; high-frequency (HF) filter, 70 Hz; low-frequency (LF) filter, 1 Hz. Continuous generalized slowing in the delta range was noted.

A 69-year-old female with a history of hypertension, migraine and depression reported worsening unsteadiness, vertigo, and headaches. She also noticed jerks of the upper extremities and random episodes of eye-shaking. At the physical exam, patient had ocular opsoclonus and a slight wide-based gait. Brain magnetic resonance imaging (MRI) was normal. She was admitted to the hospital for further evaluation. Cerebrospinal fluid (CSF) came back positive for ANNA-2. Due to the presence of opsoclonus and myoclonus at the physical exam, opsoclonus and myoclonus syndrome (OMS) secondary to ANNA-2 was diagnosed. She finished solumedrol pulses, intravenous immunoglobulin (IVIG), and prednisone taper with mild improvement in her symptoms. Whole-body positron emission tomography showed a left axillary large lymph node whose biopsy showed metastatic invasive ductal breast carcinoma. She underwent a left modified radical breast mastectomy. Patient completed chemotherapy and adjuvant radiation therapy. Symptoms almost resolved postoperatively; mild vertigo persisted. The following year, patient was diagnosed with right ocular melanoma. Right eye was enucleated. A few months later, metastasis to the liver and L1 vertebra were found. She was started on checkpoint inhibitors (ipilimumab/nivolumab). One month later, she had four generalized tonic-clonic seizures at home without recovery of consciousness. In the emergency room, she was intubated for airway protection, sedated with propofol, loaded with levetiracetam 20 mg/kg, and started on maintenance with levetiracetam 1000 mg twice a day plus lacosamide 100 mg twice a day. Physical examination was remarkable for left eye opsoclonus (right eye was prosthetic). She was admitted to the neurological intensive care unit. Brain MRI did not show relevant findings; only enhancement of the right eye sheath (post-surgical site of right ocular melanoma). Video electroencephalogram showed generalized continuous slowing (Figure 1). CSF analysis showed pleocytosis with a predominance of lymphocytes. Further workup is detailed in Table 1. For possible recurrence of paraneoplastic encephalitis, three consecutive pulses of solumedrol were given, followed by IVIG (2 g/kg) and prednisone taper for one month. CSF paraneoplastic panel was positive for ANNA-2 with lower titers than the previous panel (Table 1). Simultaneously, the patient had a new-onset thyroid disorder (antithyroglobulin elevated in serum). The patient was extubated. She was alert and oriented in three spheres without motor deficits. Opsoclonus was still present but decreased in frequency. She was discharged to rehabilitation. Oncological treatment was on hold until functional recovery. Months later, she was admitted due to sepsis and deceased.

**Table 1:** Cerebrospinal fluid tests Note that intravenous immunoglobulin was started before receiving the result of the paraneoplastic panel (it was sent out). Abbreviations: ANNA, anti-neuronal nuclear antibody; AGNA, anti-glial nuclear antibody; Ab, antibody; CRM, collapsin response mediator protein; IgG, immunoglobulin G; PCA, Purkinje cytoplasmic antibody

Chemistry and Cytology	Test Results
Red Blood Cells	<2/µL
Total Nucleated Cells	46/ µL
Glucose	100 mg/dl
Lymphocytes	38/ µL
Protein	119 mg/dl
Infectious workup (PCR)	
Meningitis and encephalitis panel (*Escherichia coli* K1, *Haemophilus influenzae*, *Listeria monocytogenes*, *Neisseria meningitidis*, *Streptococcus agalactiae*, *Streptococcus pneumoniae*, cytomegalovirus, enterovirus, herpes simplex virus (HSV) 1/2/6, human parechovirus, varicella zoster virus (VZV), *Cryptococcus neoformans/gattii*)	Negative
New York State encephalitis panel (Lyme disease, Powassan virus, West Nile virus, Eastern equine encephalitis virus, and St. Louis encephalitis virus)	Negative
Flux cytometry	normal
Histopathology	no atypical cells
Paraneoplastic panel	
ANNA-2	Positive (1:16)
AGNA-1	Negative
Amphiphysin Ab	Negative
ANNA-1	Negative
ANNA-3	Negative
CRM-5-IgG	Negative
PCA-1	Negative
PCA-2	Negative
PCA-Tr	Negative

## Discussion

The patient presented with recurrent paraneoplastic encephalitis due to ANNA-2. In the first episode, OMS was the trigger of the oncological work-up and, subsequently, breast cancer was diagnosed. In the second episode (three years ahead), the patient presented with NORSE. Opsoclonus was seen at the physical exam. 

Recurrent paraneoplastic opsoclonus syndrome is rare [5]. At the time of admission, the patient had a diagnosis of two different types of cancers: breast cancer and ocular melanoma. OMS has been described in melanoma cases rarely [5,6]. ANNA-2 is mostly associated with breast cancer [7,8], and recurrent paraneoplastic encephalitis has been reported in breast cancer [9]. 

The patient presented with NORSE. ANNA-2 is part of the suggested testing for this condition [10]. This antibody immunoreacts with Purkinje cells and CNS neuronal cells [11,12]. In the latter, the immunoreaction has been found in the nucleolus, nucleus but not nucleolus, and in the cytoplasm [12]. A review reported neuronal loss, gliosis, inflammation changes, and B-cell and T-cell infiltration, suggesting an antibody-mediated mechanism [13]. 

Interestingly, there was a temporal relation between the initiation of the symptoms and the immune checkpoint inhibition therapy (one month later). Per oncological guidelines, encephalopathy as a side effect of immune checkpoint inhibition therapy is a diagnosis of exclusion [14]. The fact that the patient had ANNA-2 was positive in CSF ruled out the diagnosis of encephalopathy as a side-effect of the immunotherapy per definition. Having said that, this therapy may have precipitated NORSE. Several mechanisms have been reported in the association of immune checkpoint inhibition therapy and status epilepticus: increased immune cell infiltration generating an inflammatory environment with increased perilesional edema and/or release of proconvulsive cytokines and a possible generation of autoantibody-mediated mechanism [15]. These cases responded relatively well to treatment with steroids or the discontinuation of immune checkpoint therapy alone [15]. To support the relevance of the side effect of this therapy, the patient never resolved her symptom of opsoclonus completely but never had seizures until having received the therapy, and the ANNA-2 titer during the NORSE admission was lower than the first admission years prior. CSF may have been positive for ANNA-2 during those three years. Alternately, the patient may have become negative for ANNA-2 during that period. The patient had undergone left radical mastectomy, and there was no evidence of active cancer in her right breast. The presence of metastasis, which may have some molecular resemblance with the old breast primary tumor, may have been enough to generate the presence of ANNA-2 in CSF. 

As our patient had a history of paraneoplastic syndrome, she was started on IVIG after the steroids pulses. Early treatment is pivotal, and steroids in isolation are not recommended in this entity [16]. Our patient responded well neurologically but was not able to restart oncological treatment due to poor functionals status.

## Conclusions

Paraneoplastic encephalitis due to ANNA-2 could relapse after years, associated with NORSE. Immune checkpoint inhibition therapy could be a precipitating factor in a patient with a history of positive ANNA-2 and current metastatic disease. A physical exam can show opsoclonus. Early immunosuppression was associated with a prompt discharge to rehabilitation.
